# Recurrent malignant peripheral nerve sheath tumor as a friable nodule within a scar

**DOI:** 10.1002/ccr3.2179

**Published:** 2019-09-13

**Authors:** Bart D. Wilkison, Scott R. Dalton, Yang Xia

**Affiliations:** ^1^ Department of Dermatology San Antonio Uniformed Services Health Education Consortium (SAUSHEC) San Antonio Texas; ^2^ Department of Pathology SAUSHEC San Antonio Texas

**Keywords:** keratoacanthoma, malignant peripheral nerve sheath tumor, marjolin's ulcer, neural tumors, squamous cell carcinoma

## Abstract

This case enforces the importance of a patient's medical history in assisting with the diagnosis of nonspecific cutaneous lesions. Also, other types of lesions occurring within a scar are reviewed.

## Graphical abstract

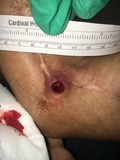

## 1 CASE REPORT

**Figure 1:**
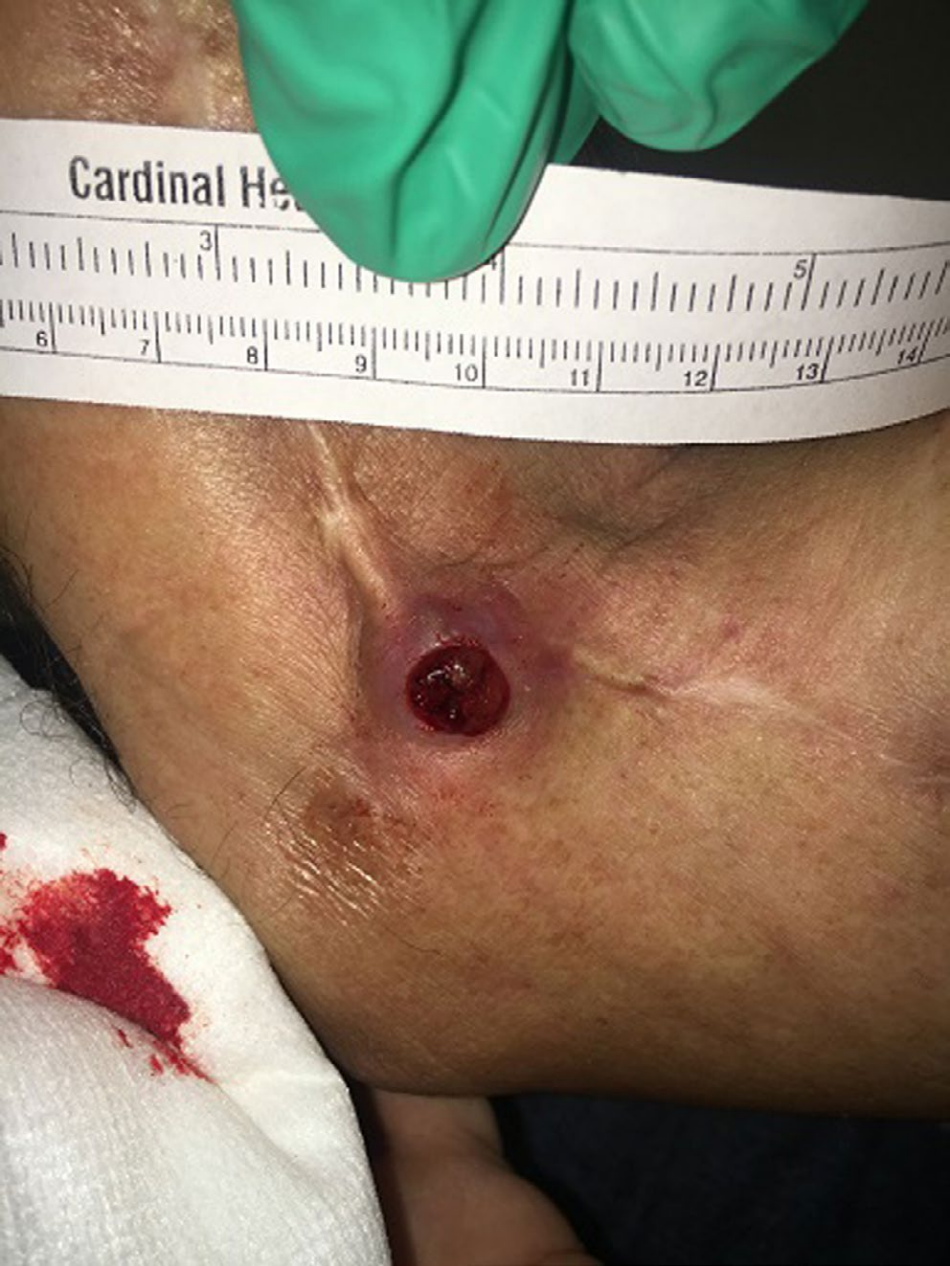
2 cm violaceous nodule with 1 cm red, friable papulonodule centrally

A 40‐year‐old male presented with a four‐week history of a rapidly growing nodule after scratching a surgical scar on his right elbow (Figure 1). In 2003, he was diagnosed with a malignant peripheral nerve sheath tumor (MPNST) on his right forearm. Treatment included radiation therapy, chemotherapy, and surgical resection with negative margins. He was disease free until 2015 when there was a recurrence over the bicep. Resection pathology showed positive margins, and the patient underwent additional radiotherapy. He denied any personal or family history of neurofibromatosis (NF). The current examination showed a 2.0 cm violaceous nodule with a central exophytic component emanating from a previous surgical scar.

**Figure 2:**
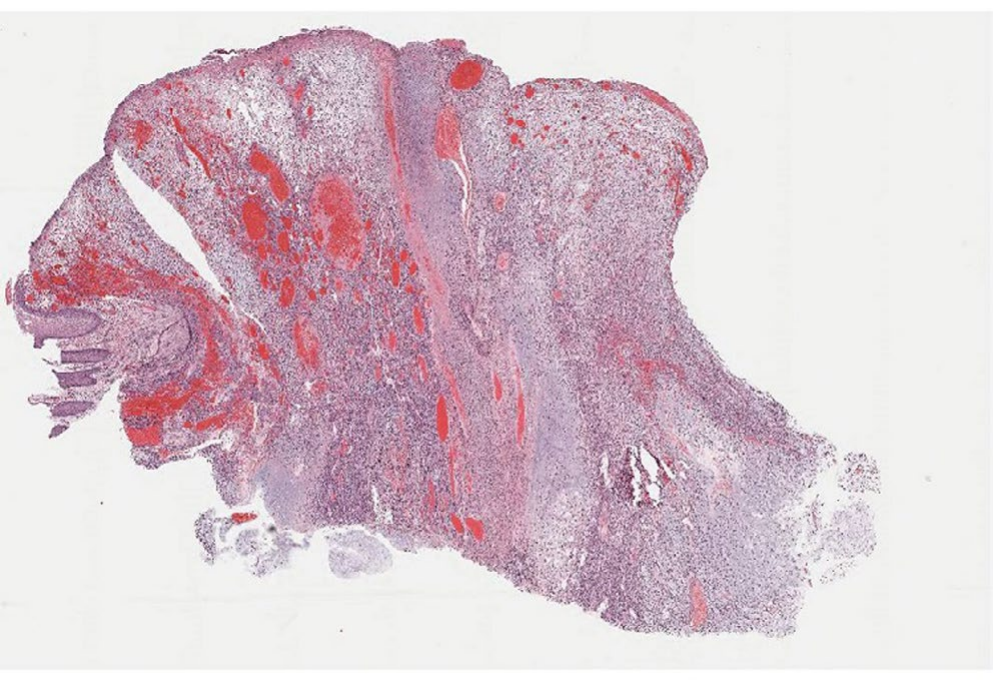
H&E Stain 2×: polypoid, ulcerated nodule with hyper‐ and hypocellular areas

**Figure 3:**
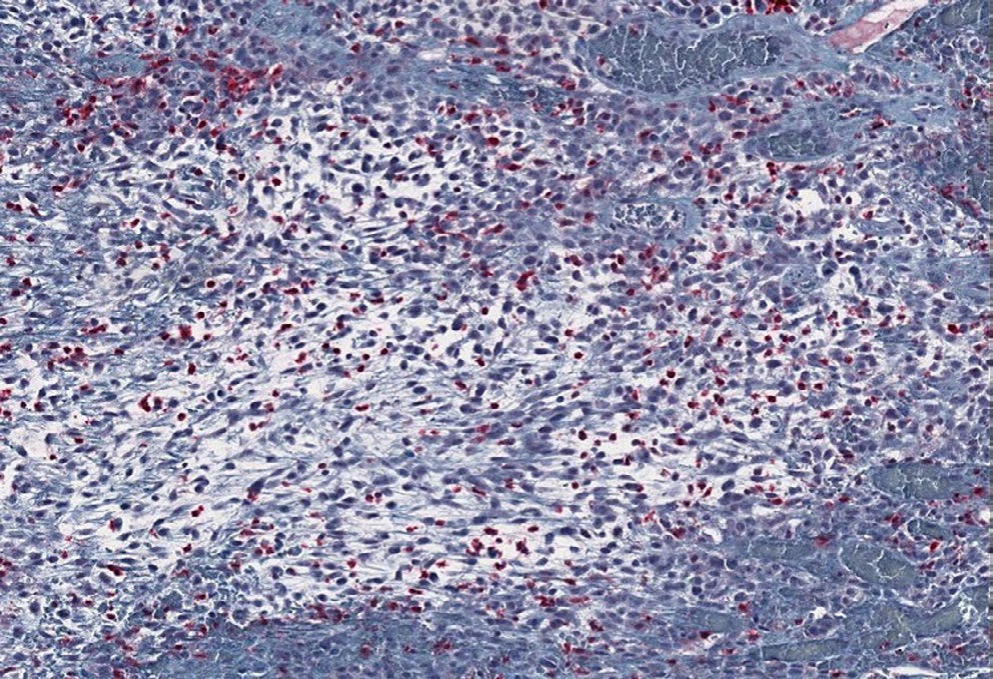
S100 Stain 20×: scattered positivity in epithelioid cells

**Figure 4:**
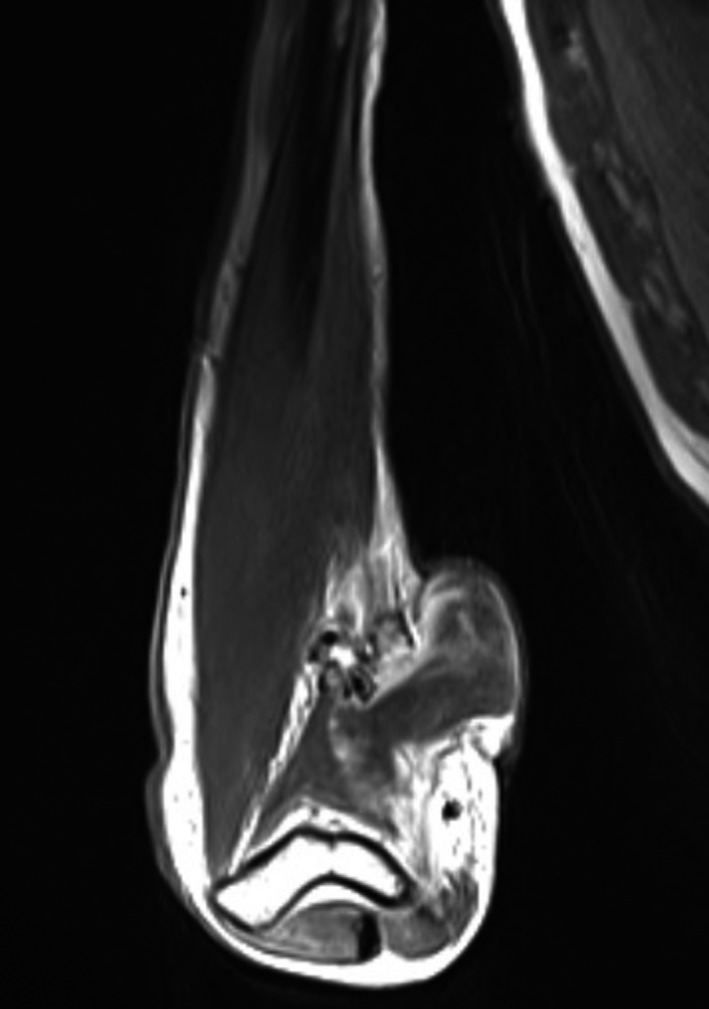
MRI, T1‐weighted image with contrast showing the patient's soft tissue mass with connection to the epidermis

Histopathologic examination showed mixed hyper‐ and hypocellular areas (Figure 2) composed of atypical spindled and epithelioid cells in a myxoid stroma. The cells demonstrated a high Ki‐67 proliferation rate, PHH3 staining with greater than 30 mitoses per high powered field, and scattered S‐100 positivity (Figure 3). MRI (Figure 4) with contrast showed a complex, dumb bell‐shaped soft tissue mass approximately 5.4 × 4.3 × 3.5 cm in size displacing the ulnar nerve medial and splaying the flexor muscles and tendons of the forearm. No metastatic lesions were found with CT and MRI. The patient elected for amputation of the right arm with surgical oncology.

Malignant peripheral nerve sheath tumor is a soft tissue tumor historically referred to as neurosarcoma or malignant schwannoma.^1^ However, studies have shown MPNST can develop from any part of the peripheral nerve sheath.^2^ MPNSTs make up 5% of all soft tissue sarcomas, and the majority of MPNSTs (50%‐66%) develop from plexiform neurofibromas in the setting of NF type I. MPNSTs arising outside of NF are typically seen in adults aged 20‐50. The tumor presents as a slowly enlarging soft tissue mass, and most commonly found on the extremities associated with pain, paresthesia, and weakness. Overall‐ and disease‐specific survival rates at 5 years are estimated at 46% and 44%, respectively.^3^

Histopathologic diagnosis with H&E can be challenging as MPNSTs can resemble other neoplasms such as synovial sarcoma, epithelioid sarcoma, leiomyosarcoma, and epithelioid angiosarcoma.^4^ Immunohistochemical stains can aid in diagnosis, but specificity is lacking.^5^ S100 staining is typically positive in 50%‐90% of tumors supporting its nerve sheath origin. When S‐100 is used in conjunction with nestin, there is a higher predictive value for correctly diagnosing MPNSTs.^6^ Positive Ki‐67 and p53 suggest a poorer prognosis. Most MPNSTs are aggressive with high rates of recurrence and distant metastasis. Surgical resection is standard of care, but chemotherapy and radiotherapy are adjunctive.

Also considered were pyogenic granuloma (PG), squamous cell carcinoma (Marjolin's ulcer), and keratoacanthoma (KA). PG's are rapidly growing papulonodules. However, the pathological results failed to show prominent lobular proliferations of ectatic vessels, superficial crusting, or epidermal collarette.^7^ KAs are not traditionally associated with scars and are usually seen in older patients. KAs are crateriform papulonodules with a central keratinous plug. KAs show squamous differentiation, but not spindle cells. Marjolin's ulcers are carcinomas (most frequently squamous cell, but basal cell has also been reported) within a previous scar or chronic wound. The majority of cases are from the lower extremities and develop from previous burn scars with an average latency of 11 years.^8^ In the current case, the relatively small, well‐circumscribed lesion on the upper extremity with the finding of spindle cells on pathology argued against this diagnosis.

## 2 CONCLUSION

Malignant peripheral nerve sheath tumor is a rare entity, and other entities associated with rapid growth and scars should be considered in the differential diagnosis of a patient with a history of sarcoma and previous radiation therapy. Histopathologic findings and immunohistochemical stains can be helpful. In this case, the diagnosis of a recurrent peripheral nerve sheath tumor was made after taking into account the patient's history, the clinical presentation, and the histopathological findings.

## CONFLICT OF INTEREST

The authors report no conflict of interest.

## AUTHOR CONTRIBUTIONS

BDW: served as the primary author, saw the patient in clinic and managed his care and is responsible for the literature review and construction of the manuscript. SRD: served as the dermatopathologist on the case and was responsible for the histopathological work‐up and final diagnosis. YX: served as the senior author managing the construction and edits of the manuscript and guiding the primary author through the submission process.
